# AI enhanced diagnostic accuracy and workload reduction in hepatocellular carcinoma screening

**DOI:** 10.1038/s41746-025-01892-9

**Published:** 2025-08-02

**Authors:** Rui-Fang Lu, Chao-Yin She, Dan-Ni He, Mei-Qing Cheng, Ying Wang, Hui Huang, Ya-Dan Lin, Jia-Yi Lv, Si Qin, Ze-Zhi Liu, Zhi-Rong Lu, Wei-Ping Ke, Chao-Qun Li, Han Xiao, Zuo-Feng Xu, Guang-Jian Liu, Hong Yang, Jie Ren, Hai-Bo Wang, Ming-De Lu, Qing-Hua Huang, Li-Da Chen, Wei Wang, Ming Kuang

**Affiliations:** 1 Department of Medical Ultrasonics, Institute of Diagnostic and Interventional Ultrasound, MedAI Collaborative Lab, Ultrasomics Artificial Intelligence X-Lab, The First Affiliated Hospital of Sun Yat-sen University, Guangzhou, China; 2 School of Artificial Intelligence, Optics and Electronics (iOPEN), Northwestern Polytechnical University, Xian, China; 3 Department of Medical Ultrasonics, The Seventh Affiliated Hospital of Sun Yat-Sen University, Shenzhen, China; 4 Department of Medical Ultrasound, The First Affiliated Hospital of Guangzhou Medical university, Guangzhou, China; 5 Department of Medical Ultrasound, The First Affiliated Hospital of Guangxi Medical University, Nanning, China; 6 Department of Medical Ultrasonics, The Sixth Affiliated Hospital, Sun Yat-sen University, Guangzhou, China; 7 Department of Medical Ultrasonics, Sanshui District People’s Hospital, Foshan, China; 8 Department of Medical Ultrasound, West China Xiamen Hospital of Sichuan University, Xiamen, China; 9 Department of Medical Ultrasonics, The Third Affiliated Hospital of Sun Yat-Sen University, Guangzhou, China; 10 School of Mechanical Engineering, Tongji University, Shanghai, China

**Keywords:** Medical research, Health care, Medical imaging

## Abstract

Hepatocellular carcinoma (HCC) ultrasound screening encounters challenges related to accuracy and the workload of radiologists. This retrospective, multicenter study assessed four artificial intelligence (AI) enhanced strategies using 21,934 liver ultrasound images from 11,960 patients to improve HCC ultrasound screening accuracy and reduce radiologist workload. UniMatch was used for lesion detection and LivNet for classification, trained on 17,913 images. Among the strategies tested, Strategy 4, which combined AI for initial detection and radiologist evaluation of negative cases in both detection and classification phases, outperformed others. It not only matched the high sensitivity of original algorithm (0.956 vs. 0.991) but also improved specificity (0.787 vs. 0.698), reduced radiologist workload by 54.5%, and decreased both recall and false positive rates. This approach demonstrates a successful model of human-AI collaboration, not only enhancing clinical outcomes but also mitigating unnecessary patient anxiety and system burden by minimizing recalls and false positives.

## Introduction

Primary liver cancer ranks as the sixth most common cancer globally and is the third leading cause of cancer-related mortality worldwide^1^. Hepatocellular carcinoma (HCC) is the predominant form of primary liver cancer. Screening for HCC in high-risk populations is critical for effective treatment and prognosis^2–4^.

The American Association for the Study of Liver Diseases guidance recommends ultrasound as the primary method for HCC screening due to its widespread availability^3,4^. According to the original algorithm, ultrasound screening images should be read by radiologists, and detected lesions ≥1 cm necessitate a recall for further evaluation with CT or MRI examinations. However, radiologists encounter difficulties differentiating between benign and malignant lesions ≥1 cm, underscoring the necessity for enhanced screening approaches. Moreover, the rising number of ultrasound screening, driven by their convenience and low cost, significantly increases the clinical workload for radiologists^5^. This surge may not only amplifies patient anxiety due to false positives and unnecessary recalls, but also risks disease progression from missed diagnoses^6^.

To address these challenges, incorporating artificial intelligence (AI) into the HCC screening process offers a promising solution. Research indicates that AI assistance in screening colorectal cancer, lung nodules and breast cancer can improve diagnostic accuracy and reduce the workload of radiologists^7–13^. The integration strategy and collaborative dynamics between AI and radiologists significantly impacts the effectiveness of screenings. Therefore, it is essential to develop a suitable AI-assisted screening process that not only improves the sensitivity and specificity of HCC screening but also reduces the workload of radiologists.

**Fig. 1:**
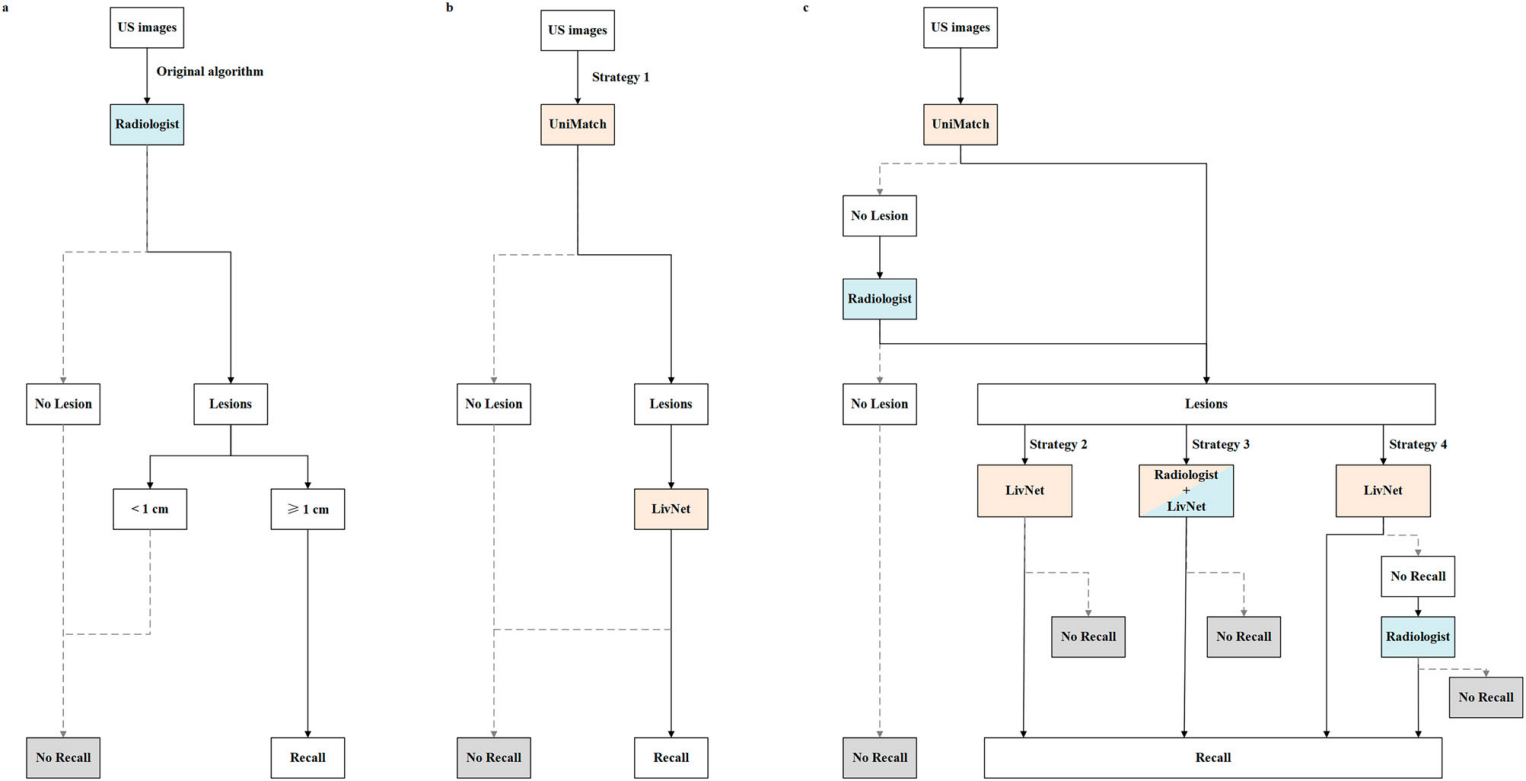
Flowchart of the original algorithm and the four human-artificial intelligence (AI) interaction strategies. Blue boxes indicate instances where the radiologist is engaged in the work. Orange boxes indicate instances where AI completely replaces the radiologist. A box that is half blue and half orange indicates instances where AI assists the radiologist, allowing them to autonomously decide whether to adopt or disregard AI suggestions. Gray boxes indicate endpoints that require no recall. **a** The original algorithm: radiologists performed lesion detection and classification on images, deciding whether to recall or no recall. **b** Strategy 1 (Stand-Alone AI): UniMatch was used in lesion detection and LivNet was used in lesion classification to determine whether to recall or not. **c** Strategies 2, 3, and 4 are identical in the lesion detection. UniMatch was employed to detect lesions. If no lesion was detected, the image would be further evaluated by radiologists. After that, images without a lesion require no recall. Images with lesions detected either by radiologists or UniMatch are then classified using different methods according to the various strategies. In Strategy 2 (AI as a triage tool with radiologist read negative cases in lesion detection), images with detected lesions were classified by LivNet. Benign lesions did not require recall, whereas malignancy did. In Strategy 3 (AI as a triage tool and radiologist’s aid), images with detected lesions were classified by radiologists assisted by LivNet. In Strategy 4 (AI as a triage tool with radiologist read negative cases in lesion detection and classification), images with detected lesions were classified by LivNet. Lesions assigned as malignant required recall, while those assigned as benign were further evaluated by radiologists to determine the necessity of recall was, aiming to ensure a high sensitivity of classification.

With AI’s potential to mimic the benefits of double reviews by providing a consistent, unbiased second opinion, our study introduces an AI-enhanced screening procedure for HCC, consisting of two innovative components: UniMatch, designed to detect liver lesions, and LivNet, engineered to classify lesions for appropriate recall actions. These components provide a robust mechanism to address the absence of double reading in ultrasound-based HCC screening. We explore the integration of AI as a triage tool into the HCC screening process and propose four human-AI interaction strategies (Fig. 1). Our aim is to evaluate these strategies to determine whether AI-assisted methods can optimize screening performance and reduce the workload of radiologists.

## Results

### Population characteristics

**Fig. 2:**
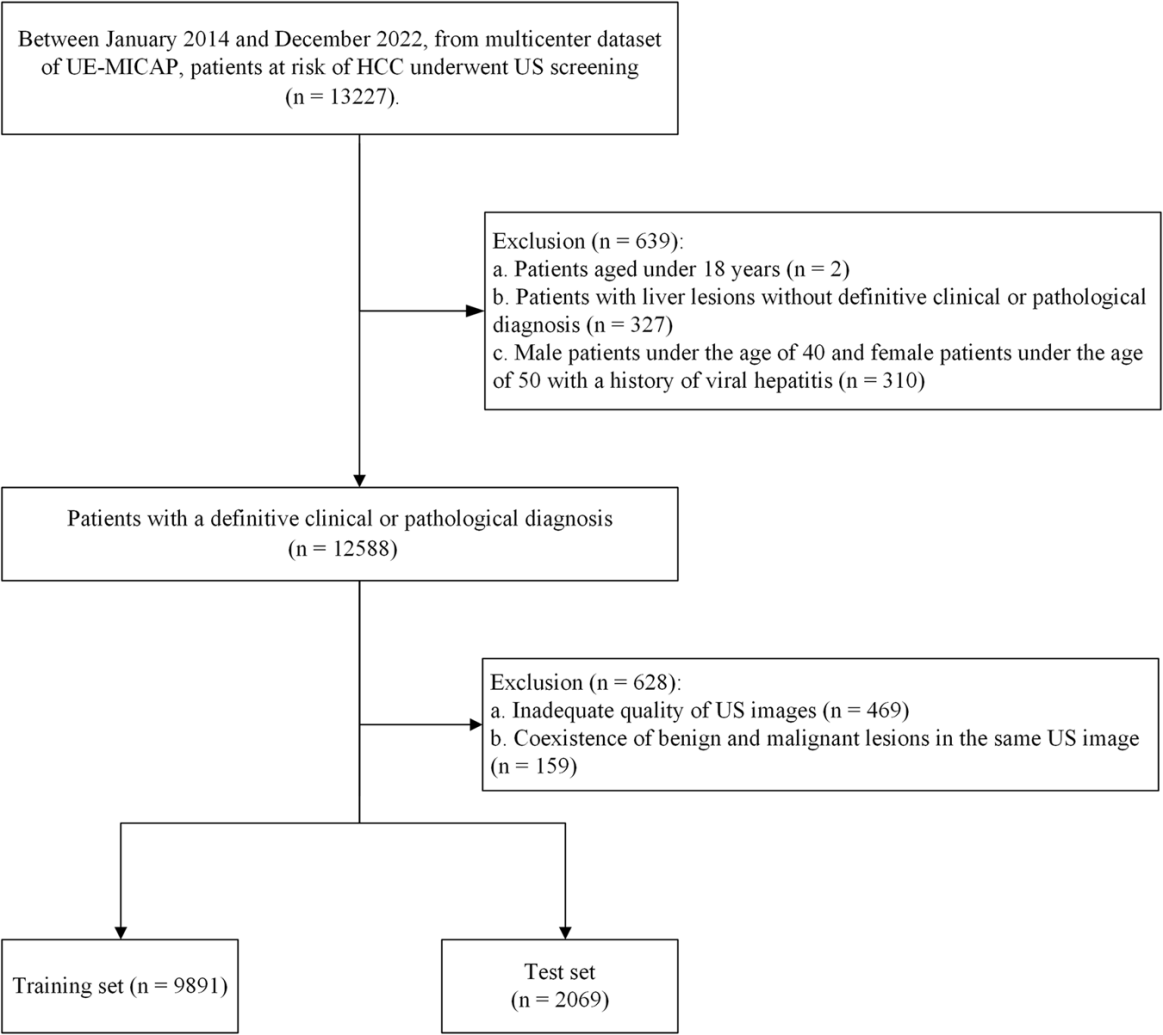
Data collection process for model training and testing in human-AI interaction strategies. Flowchart illustrates the data collection process for the model training and test for human-AI interaction strategies. The study initially involved 13,227 patients at risk of hepatocellular carcinoma who underwent ultrasound screening. After 1267 patients were excluded, the final patients were divided into a training set of 9891 and a test set of 2069.

A total of 25,629 images of 13,227 patients with ultrasound screening were initially included in the study. 2 patients were excluded due to being under 18 years of age. 327 patients were excluded due to a lack of definitive clinical or pathological diagnosis. 310 patients were excluded due to being male under 40 years old or female under 50 years old with a history of viral hepatitis. 469 patients were excluded due to inadequate quality of images. 159 patients were excluded due to the coexistence of benign and malignant lesions in the same images (Fig. 2). Finally, training set for lesion detection included 17,913 images from 9891 screenings (mean age, 44.8 years±14.7), training set for lesion classification consisted of 11 244 images of 6305 screenings (mean age, 48.2 years±13.9), and test set included 4021 images of 2069 screenings (mean age, 54.0 years±10.7) (Table 1).

**Table 1:** Summary of demographic characteristics

Characteristic	Training set for lesion detection	Training set for lesion classification	Test set
Number of screenings	9891	6305	2069
Number of images	17,913	11,244	4021
Mean age (mean ± SD)	44.8 ± 14.7	48.2 ± 13.9	54.0 ± 10.7
Number of screenings by sex			
Male	5284 (53.4)	3342 (53.0)	1706 (82.5)
Female	4607 (46.6)	2963 (47.0)	363 (17.5)
Number of images by sex			
Male	10,102 (56.4)	6064 (53.9)	3330 (82.8)
Female	7811 (43.6)	5180 (46.1)	691 (17.2)
Number of images			
Lesions	14,046 (78.4)	11,244 (100)	3020 (75.1)
Benign	10,285 (57.4)	8198 (72.9)	731 (18.2)
Malignant	3761 (21.0)	3046 (27.1)	2289 (56.9)
No lesions	3867 (21.6)	0 (0)	1001 (24.9)
Size of lesions			
<1 cm	1771 (12.6)	1402 (12.5)	228 (7.5)
≥1 cm	12,275 (87.4)	9842 (87.5)	2792 (92.5)

*SD* standard deviations. Unless otherwise indicated, data are number of images; data in parentheses are percentages.

### Performance of models for lesion detection and classification

In test set, UniMatch, a model specifically developed for lesion detection, achieved a sensitivity of 0.941 (95%CI 0.932–0.949), a specificity of 0.833 (0.808–0.855), an accuracy of 0.914 (0.905–0.923, 3676 of 4021 images), and an AUC of 0.887 (0.876–0.896). A total of 180 images without lesions were erroneously classified as containing lesions, while 165 images with lesions were incorrectly classified as lesion-free. The statistical results of lesion sizes for images with lesions that were incorrectly classified as lesion-free are presented in Supplementary Table No. 1. The performance of UniMatch on the test set is illustrated in Supplementary Fig. 1.

The cutoff value for LivNet, a model specifically developed for lesion classification, was set at 0.2, which was chosen taking into account radiologists’ recall rate in order to potentially maintain sensitivity. The sensitivity, specificity, accuracy, and AUC of LivNet were 0.891 (95% CI 0.877–0.903), 0.783 (0.762–0.802), 0.844 (0.832–0.855) and 0.837 (0.825–0.848), respectively. The visualization of heatmaps by LivNet is presented in Supplementary Fig. 2.

Both the training and test sets underwent a de-marker process, as detailed in the Development of De-markers Model section of the Methods. We additionally assessed the performance of UniMatch and LivNet on a version of the test set containing markers, with results reported in Supplementary Table No. 2 and Supplementary Data 1. Compared to the marker-free test set, test set containing markers led to significantly improved sensitivity, accuracy, and AUC for UniMatch, with a slight decrease in specificity (*p* < 0.001), likely due to marker guidance facilitating target localization. In contrast, LivNet showed increased sensitivity but decreased specificity, accuracy, and AUC (*p* < 0.001), possibly because it was trained on marker-free images and the presence of markers obscured critical image features, impairing classification performance.

### Performance of screening for human-AI interaction strategies

**Fig. 3:**
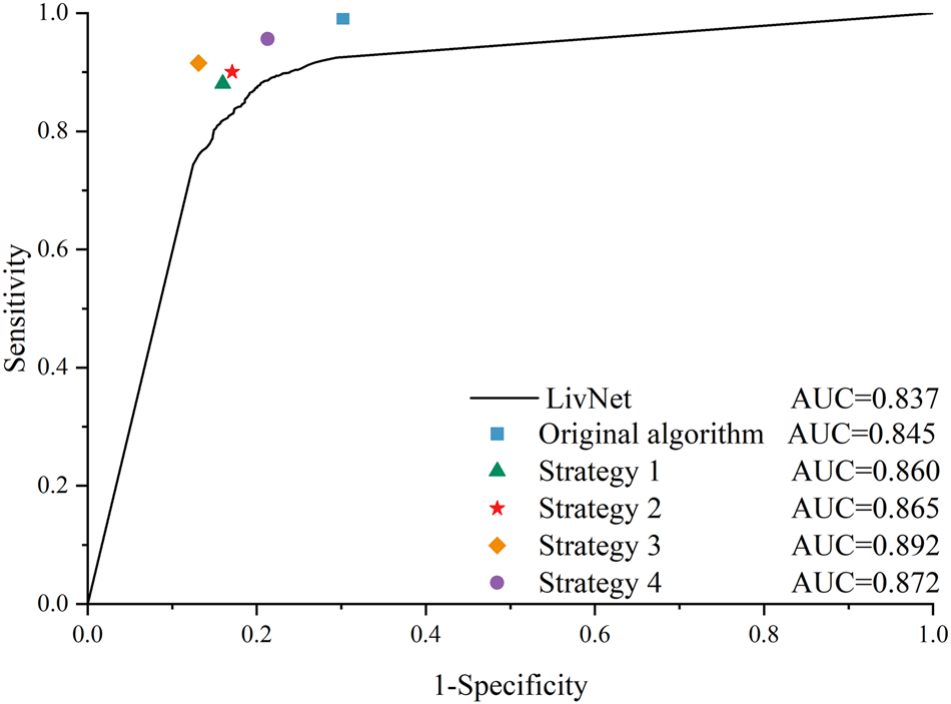
Performance of original algorithm and four Human-AI interaction strategies in test set. The Receiver Operating Characteristic (ROC) curves compared the performance of different AI-enhanced strategies for hepatocellular carcinoma ultrasound screening. The black line represents the ROC curve for LivNet, used as a baseline for comparison. Each symbol on the graph represents a different strategy: the square (blue) for the original algorithm, the triangle (green) for Strategy 1, the star (red) for Strategy 2, the diamond (orange) for Strategy 3, and the circle (purple) for Strategy 4. The area under the curve (AUC) for each strategy demonstrated the varying levels of diagnostic accuracy: LivNet (AUC = 0.837), Original algorithm (AUC = 0.845), Strategy 1 (AUC = 0.860), Strategy 2 (AUC = 0.865), Strategy 3 (AUC = 0.892), and Strategy 4 (AUC = 0.872).

The comparison of the original algorithm with the four strategies is presented in Table 2. The original algorithm achieved an AUC of 0.845 (95% CI 0.833–0.856), a sensitivity of 0.991 (0.987–0.995), and a specificity of 0.698 (0.676–0.720). Compared to the original algorithm, Strategy 1, Strategy 2, and Strategy 3 each achieved a higher AUC (0.860 [0.849–0.871, *p* < 0.001], 0.865 [0.854–0.875, *p* < 0.001], and 0.892 [0.886–0.898, *p* < 0.001], respectively), lower sensitivity (0.880 [0.866–0.893, inferiority, *p* < 0.001], 0.900 [0.887–0.912, inferiority, *p* < 0.001], and 0.916 [0.909–0.922, inferiority, *p* < 0.001], respectively), and higher specificity (0.840 [0.822–0.857, superiority, *p* < 0.001], 0.829 [0.811–0.847, superiority, *p* < 0.001], and 0.869 [0.859–0.878, superiority, *p* < 0.001], respectively). Strategy 4 resulted in noninferior sensitivity (0.956 [0.951–0.961, noninferiority, *p* < 0.001]) and superior specificity (0.787 [0.776–0.799, superiority, *p* < 0.001]) with a higher AUC (0.872 [0.865–0.877, *p* < 0.001]) (Fig. 3).

**Table 2:** Performance of original algorithm and different human-AI interaction strategies

	AUC	*P* value	Sensitivity	Rate difference	*P* value	Specificity	Rate difference	*P* value	Accuracy	P value
Original algorithm	0.845 (0.833–0.856)	..	0.991 (0.987–0.995)	..	..	0.698 (0.676–0.720)	..	..	0.865 (0.854–0.875)	..
Strategy 1	0.860 (0.849–0.871)	<0.001	0.880 (0.866–0.893)	−0.111 (−0.125–−0.097)	<0.001	0.840 (0.822– 0.857)	0.142 (0.114–0.170)	<0.001	0.863 (0.852–0.873)	0.795
Strategy 2	0.865 (0.854–0.875)	<0.001	0.900 (0.887–0.912)	−0.091 (−0.104–−0.078)	<0.001	0.829 (0.811–0.847)	0.131 (0.103–0.159)	<0.001	0.870 (0.859–0.880)	0.532
Strategy 3	0.892 (0.886–0.898)	<0.001	0.916 (0.909–0.922)	−0.076 (−0.083–−0.068)	<0.001	0.869 (0.859–0.878)	0.171 (0.147–0.194)	<0.001	0.895 (0.890–0.901)	<0.001
Strategy 4	0.872 (0.865–0.877)	<0.001	0.956 (0.951–0.961)	−0.035 (−0.041–−0.029)	<0.001	0.787 (0.776–0.799)	0.089 (0.065–0.113)	<0.001	0.883 (0.877–0.889)	<0.001

AUC area under the receiver operating characteristic curve. Data in parentheses are 95% CIs. The *P* values were computed for comparisons between original algorithm and different strategy. The rate differences were computed for sensitivity and specificity between original algorithm and different strategy. All *P* values were Bonferroni multiple hypothesis corrected.

In the test set, all 4021 images required evaluation by a radiologist according to the original guidance. Compared to the original algorithm (4021 images), Strategy 1, which relied solely on AI analysis, dramatically reduced the workload by 100% (4021 of 4021 images, *p* < 0.001), Strategy 2 reduced by 75.5% (3035 of 4021 images, *p* < 0.001), whereas Strategy 3 increased by 4.10% (4186 images, *p* < 0.001). Strategy 4 reduced by 54.5% (2192 of 4021 images, *p* < 0.001) (Table 3).

**Table 3:** Workload and recall rate of original algorithm and different human-AI interaction strategies

	Workload	Workload reduction (%)	*P* value	Recall rate	*P* value	False positive rate	*P* value
Original algorithm	4021	..	..	0.694 (0.680–0.709)	..	0.302 (0.280–0.324)	..
Strategy 1	0	100.0 (99.9–100)	<0.001	0.570 (0.555–0.585)	<0.001	0.160 (0.143–0.178)	<0.001
Strategy 2	986	75.5 (74.1–76.8)	<0.001	0.586 (0.571–0.601)	<0.001	0.171 (0.153–0.189)	<0.001
Strategy 3	4186	−4.1 (−4.8–−3.5)	<0.001	0.578 (0.569–0.587)	<0.001	0.131 (0.122–0.141)	<0.001
Strategy 4	1829	54.5 (53.0–56.1)	<0.001	0.636 (0.627–0.645)	<0.001	0.213 (0.202–0.224)	<0.001

Data in parentheses are 95% CIs. Workload data are numbers of images. Workload reduction was computed as the difference in workload between the original algorithm and different strategies, divided by the workload of the original algorithm. The *P* values were computed for comparisons between original algorithm and different strategy. All *P* values were Bonferroni multiple hypothesis corrected.

While the original algorithm had the highest recall rate of 0.694 (95% CI 0.680–0.709), it also exhibited the highest false positive rate of 0.302 (0.280–0.324) among all strategies. In contrast, Strategy 1 had a recall rate of 0.570 (0.555–0.585, *p* < 0.001) and a false positive rate of 0.160 (0.143–0.178, *p* < 0.001), Strategy 2 exhibited a recall rate of 0.586 (0.571–0.601, *p* < 0.001) and a false positive rate of 0.171 (0.153–0.189, *p* < 0.001), Strategy 3 achieved a recall rate of 0.578 (0.569–0.587, *p* < 0.001) and the lowest false positive rate 0.131 (0.122–0.141, *p* < 0.001), and Strategy 4 had a recall rate of 0.636 (0.627–0.645, *p* < 0.001) and a false positive rate of 0.213 (0.202–0.224, *p* < 0.001) (Table 3).

### Uncertainty of different strategies

**Fig. 4:**
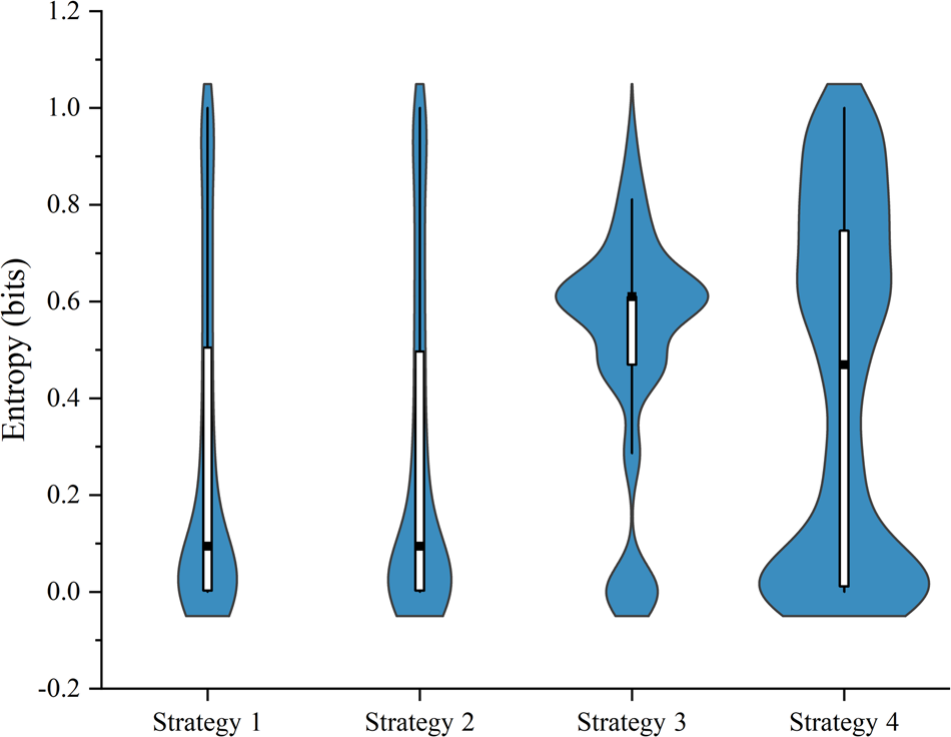
Entropy of incorrect judgments across four strategies in test set. The violin plot depicts the distribution of entropy for incorrect judgments across four strategies on the test set. The width of the violin plot at each point corresponds to the density of the data at that value. Boxes represent the 25th–75th percentiles, the whiskers indicate the minimum and maximum values, and the solid black squares represent the medians.

We calculated the Shannon entropy of different strategies during the classification stage and used it as the uncertainty of strategy. A higher entropy value indicates a higher uncertainty in the prediction results. We found that Strategy 3 (median: 0.286 bits, IQR 0.609 bits) and Strategy 4 (median: 0.000 bits, IQR 0.532 bits) exhibited higher entropy in their predictions compared to Strategy 1 (median: 0.000 bits, IQR 0.045 bits) and Strategy 2 (median: 0.000 bits, IQR 0.052 bits). For all comparisons between the strategies, except those involving Strategy 1 and Strategy 2 (*p* = 0.1988), the *p* values were below the adjusted *p* value (0.05/6), indicating significant differences. Furthermore, we calculated the entropy values of different strategies in cases of incorrect judgments and found that, relative to Strategy 1 (number of errors 487, median of entropy 0.094 bits, IQR of entropy 0.501 bits) and Strategy 2 (number of errors 524, median of entropy 0.094 bits, IQR of entropy 0.495 bits), Strategy 3 (average number of errors 421, median of entropy 0.610 bits, IQR of entropy 0.141 bits) and Strategy 4 (average number of errors 470, median of entropy 0.469 bits, IQR of entropy 0.737 bits) reduced excessive confidence in diagnostic errors without compromising diagnostic accuracy (Fig. 4). For all comparisons between the strategies, except those involving Strategy 1 and Strategy 2 (*p* = 0.8866), the *p* values were below adjusted *p* value. On the other hand, in the cases with very low entropy values (entropy <0.1), where AI or radiologist made decisions with high certainty, the error rate of Strategy 1 to Strategy 4 is 10.33% (246 of 2381 images), 10.64% (266 of 2499 images), 4.49% (191 of 4253 images), and 7.96% (482 of 6052 images), respectively. This indicates that Strategy 3 and Strategy 4 demonstrate higher reliability in highly deterministic decision-making. For all comparisons between the strategies, except those involving Strategy 1 and Strategy 2 (*p* = 0.722), the *p* values were below adjusted *p* value.

### Subgroup analysis

The test set with lesions was divided into two subgroups: the small lesion group (lesions smaller than 1 cm; *n* = 228; mean age 54.00 years±10.80), and the large lesion group (lesions larger than 1 cm; *n* = 2792; mean age 55.57 years±11.02). In the small lesion group, Strategy 4 achieved improved sensitivity (0.833 [95% CI 0.715–0.918]) compared to other strategies of 0.700 ([0.457–0.881], *p* = 0.197), 0.750 ([0.509–0.913], *p* = 0.408), and 0.500 ([0.368–0.632], *p* < 0.001), respectively. And Strategy 4 had lower specificity (0.702 [0.664–0.738]) compared to other strategies of 0.803 ([0.742–0.855], *p* = 0.005), 0.769 ([0.706–0.825], *p* = 0.062), and 0.808 ([0.775–0.838], *p* < 0.001), respectively. In the large lesion group, Strategy 4 showed improved sensitivity (0.957 [95% CI 0.952–0.962]) compared to other strategies of 0.882 ([0.868–0.895], *p* < 0.001), 0.902 ([0.889–0.914], *p* < 0.001), and 0.919 ([0.912–0.926], *p* < 0.001), respectively. And Strategy 4 had lower specificity (0.528 [0.503–0.553]) compared to other strategies of 0.663 ([0.621–0.704], *p* < 0.001), 0.641 ([0.598–0.682], *p* < 0.001), and 0.642 ([0.618–0.666], *p* < 0.001), respectively (Table 4).

**Table 4:** Performance of different human-AI interaction strategies in two subgroups

	AUC	*P* value	Sensitivity	Rate difference (%)	*P* value	Specificity	Rate difference (%)	P value	Accuracy	P value
The Small Lesion Group										
Strategy 1	0.751 (0.690–0.806)	0.789	0.700(0.457–0.881)	0.133 (−0.089–0.355)	0.197	0.803 (0.742–0.855)	−0.100 (−0.166–−0.037)	0.005	0.793 (0.736–0.844)	0.017
Strategy 2	0.760 (0.699–0.814)	0.890	0.750 (0.509–0.913)	0.083 (−0.129–0.295)	0.408	0.769 (0.706–0.825)	−6.731(−13.488–0)	0.062	0.768 (0.707–0.821)	0.112
Strategy 3	0.654 (0.617–0.690)	0.007	0.500 (0.368–0.632)	0.333 (0.176–0.491)	<0.001	0.808 (0.775–0.838)	−0.106(−0.153–−0.058)	<0.001	0.781 (0.748–0.811)	0.004
Strategy 4	0.768 (0.734–0.799)	..	0.833 (0.715–0.918)	..	..	0.702 (0.664–0.738)	..	..	0.713 (0.678–0.747)	..
The Large Lesion Group										
Strategy 1	0.773 (0.757–0.788)	0.017	0.882(0.868–0.895)	0.075 (0.061–0.089)	<0.001	0.663 (0.621–0.704)	−0.136(−0.183–−0.088)	<0.001	0.841 (0.827–0.854)	<0.001
Strategy 2	0.771 (0.755–0.787)	0.024	0.902(0.889–0.914)	0.056 (0.042–0.069)	<0.001	0.641 (0.598–0.682)	−0.113(−0.161–−0.065)	<0.001	0.853 (0.839–0.866)	0.001
Strategy 3	0.781 (0.771–0.789)	<0.001	0.919 (0.912–0.926)	0.038 (0.030–0.046)	<0.001	0.642 (0.618–0.666)	−0.114(−0.148–−0.080)	<0.001	0.867 (0.860–0.874)	0.064
Strategy 4	0.742 (0.733–0.752)	..	0.957 (0.952–0.962)	..	..	0.528 (0.503–0.553)	..	..	0.877 (0.870–0.884)	..

AUC area under the receiver operating characteristic curve. Data in parentheses are 95% CIs. The *P* values were computed for comparisons between Strategy 4 and other strategies. All *P* values were Bonferroni multiple hypothesis corrected.

## Discussion

We simulated four different human-AI interaction strategies, emphasizing how AI can be integrated into HCC screening in different ways to reduce workload and improve specificity without compromising sensitivity. Particularly, Strategy 4 performed exceptionally well, achieving noninferiority in sensitivity and superiority in specificity compared to the original algorithm, while simultaneously reducing workload by 54.5% (2192 of 4021 images). This was accomplished by having radiologists further examined the screenings classified as no lesions by UniMatch and review the screenings considered no recall by LivNet, thereby ensuring that no suspicious screenings were missed.

The strategy of integrating AI and its collaboration with radiologists critically influences the effectiveness of screenings. Several studies have investigated how AI can reduce the workload in breast cancer screening^9–15^. Lång K et al. reported the AI-supported mammography screening, which significantly reduced the reading workload by 44.3% compared to standard double-reading, while ensuring safety metrics such as cancer detection rate (6.1 per 1000 screened participants in the AI group vs 5.9 per 1000 screened participants in the standard group), recall rate (0.042 vs. 0.045), and false positive rate (0.038 vs 0.040)^9^. Lauritzen AD et al. utilized an AI system to assess the risk of mammograms, mandating double-reading for moderate risk cases. The results maintained that compared to radiologist-based screening, the AI-assisted screening had similar sensitivity (0.697 vs 0.708) and higher specificity (0.986 vs 0.981), while reducing 0.626 of the radiologists’ workload and decreasing the false-positive rate by 0.251^13^. To our knowledge, this is the first multicenter study to compare various HCC screening strategies. Previous studies on AI applications for liver ultrasound predominantly concentrated on disease diagnosis and lesion segmentation, comparing AI and radiologist performance, utilizing AI to aid radiologists or simply developing deep learning models, neglecting how AI could be integrated into screening workflows to enhance efficiency^16–22^.

Current research indicates that substantial quality and quantity of evidence is still required before AI systems can be implemented into clinical practice^23–26^. This rationale underpinned our design of diverse strategies, where AI and radiologists were integrated at various stages, enabling us to assess the optimal utilization of AI systems while progressively building trust and acceptance. Strategy 4 employs a layered detection and decision-making approach that capitalizes on the combined strengths of AI models and radiologists. Initially, UniMatch assesses whether lesions are present in the images. Subsequently, radiologists review the images deemed lesion-free by UniMatch, compensating for potential limitations of AI such as missed small lesions, to ensure that no suspicious case is overlooked. For images with lesions identified by both the AI model and radiologists, LivNet is then utilized for lesion classification. Radiologists further review the images classified as no recall by LivNet, ensuring high sensitivity of 0.956 in the screening process. Concurrently, the high classification accuracy of the UniMatch and LivNet contributes to an improved specificity of 0.787 in the screening, reducing the likelihood of erroneous diagnoses.

Notably, by deploying AI models for initial triage and leveraging the high performance of both models, radiologists only need to review the images deemed negative by the AI models, thereby eliminating the need to examine all images. Compared to original algorithm, this approach significantly reduces the radiologists’workload by 54.5%, while simultaneously lowering the recall rate from 0.694 to 0.636, and reducing the false positive rate from 0.302 to 0.213. It reduces unnecessary additional tests, conserving valuable medical resources and lowering healthcare costs for both patients and providers. Through this multi-layered screening and decision-making process, Strategy 4 optimizes efficiency while maintaining diagnostic accuracy.

We have also addressed the uncertainty associated with different strategies. Compared to other strategies, Strategy 3 and Strategy 4 demonstrate a reduction in overconfidence regarding diagnostic errors while maintaining diagnostic accuracy. Notably, when AI or radiologists render highly certain decisions, Strategy 3 and Strategy 4 exhibit lower error rates. Particularly, Strategy 4 leverages multiple evaluations from AI and radiologists in both detection and classification processes. This approach not only identifies and corrects potential misdiagnoses but also ensures the repeated verification of potentially erroneous information.

Our study has limitations. First, it is a retrospective study, subject to potential recall bias. Although the model of UniMatch for lesion detection are well-suited for countries where sonographers perform the examinations and radiologists generate the reports, they may not be applicable in settings where the same radiologist conducts both the ultrasound examination and prepares the report, as they would already know whether the patient has liver nodules during the scanning process. Second, our limited data from eight centers, which may limit its generalizability. Each center may have different patient populations and equipment, leading to variations in the applicability of results to other institutions. To enhance the generalizability of the findings, future studies could consider expanding the sample size. Future work could also explore domain adaptation to address distributional shifts across centers, caused by differences in equipment, imaging protocols, and populations^27–30^. For instance, a recent fetal ultrasound study introduced a domain-adaptive standard plane generator to align video and image features, enhancing generalizability across data sources^31^. Incorporating these approaches in future work could further strengthen the clinical applicability and generalizability of our model. Finally, the increased workload in Strategy 3 was due to radiologists needing to re-evaluate screenings that were initially classified by UniMatch as lesion-free but later reviewed with the assistance of LivNet. This approach may not represent the most efficient strategy. In future research, the implementation of this strategy could be optimized.

In conclusion, incorporating AI into HCC screening may improve screening outcomes, including enhanced specificity as well as reduced recall rate and false positive rate. Strategy 4, in which AI initially detects lesions and subsequently triages screenings with detected lesions while radiologists review only those screenings not flagged for recall, helps to significantly alleviate the excessive workload typically faced by radiologists. Further prospective experimental validation of our results is needed.

## Methods

### Study design and participants

This retrospective, multicenter study received ethical approval from the Research Ethics Committee of each hospital: the First Affiliated Hospital of Sun Yat-sen University, the Third Affiliated Hospital of Sun Yat-sen University, the Sixth Affiliated Hospital of Sun Yat-sen University, the Seventh Affiliated Hospital of Sun Yat-sen University, the First Affiliated Hospital of Guangzhou Medical University, the First Affiliated Hospital of Guangxi Medical University, Foshan Sanshui District People’s Hospital, and West China Xiamen Hospital of Sichuan University. As the study was retrospective in nature and images used were fully anonymized, the requirement for informed consent was waived. Data for this study were retrospectively collected from multicenter dataset of Ultrasound Engineering Institute, Medical Industry Branch of China Association Plant Engineering (UE-MICAP). Among them, HCC screening images were sourced from the above eight hospitals between January 2014 and December 2022, involving a total of 11,960 screenings and 21,934 images. The inclusion criteria included: (a) patients at risk for HCC, including those with a clinical diagnosis or imaging indicative of cirrhosis, or pathological evidence of cirrhosis, (b) male patients over the age of 40 and female patients over the age of 50 with a history of viral hepatitis, and (c) patients who underwent ultrasound screening, and if a lesion was detected, the lesion was confirmed with a definitive clinical or pathological diagnosis. The exclusion criteria included: (a) age under 18 years, (b) inadequate image quality, and (c) coexistence of benign and malignant lesions in the same image, as this could compromise the AI’s ability to classify lesions accurately (Fig. 2).

### Procedures

All ultrasound examinations were conducted by an attending radiologist at each center using the same criteria after patients fasted for at least 8 h. During the screening, images of standard scan planes were stored in the Picture Archiving and Communication Systems, which included the first and second hepatic portal, hepato-renal plane, and longitudinal section of the left liver. If a lesion was detected, images of the lesion were also archived. All images were subsequently reviewed by a radiologist to exclude those with poor image quality. Additionally, YOLOv3, a vision AI model for image detection, was used to anonymize the ultrasound images by removing patient information from the ultrasound images, retaining only the fan-shaped ultrasound area^32^. To mitigate bias during image training, we developed a de-markers model specifically designed to remove measurement scales from ultrasound images.

### Development of De-markers model

**Fig. 5:**
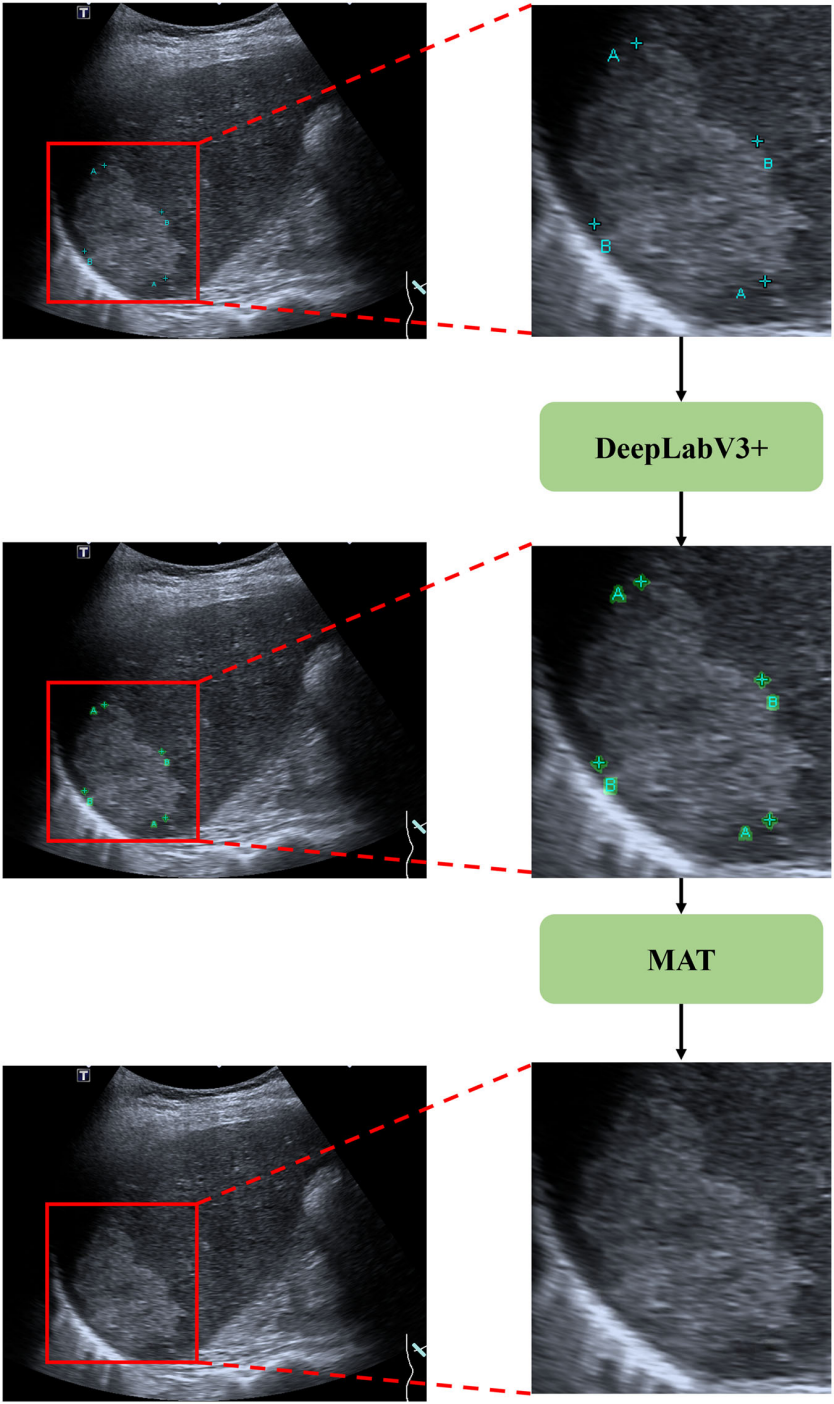
Network architecture of de-markers model. De-markers model illustrates a two-stage generative algorithm that combines a segmentation model (DeepLabv3 + ) and a Transformer-based inpainting model (MAT) to effectively remove measurement markers from ultrasound images. MAT Mask-Aware Transformer.

Measurement markers from ultrasound images are utilized for measuring the long and short diameters of tumors. These markers can interfere with the analysis and interpretation of the images, potentially leading to biases in detection models. To mitigate this problem, we have developed a two-stage generative algorithm for the removal of these markers (Fig. 5).

In the first stage, we collected pixel-wise matched pairs of ultrasound images, one with measurement markers and one without. These pairs were used to train a segmentation mode, DeepLabv3+ which accurately identifies and segments the regions containing the measurement markers^33^. This step is crucial as it allows for the precise localization of the markers within the images.

In the second stage, we utilized the Mask-Aware Transformer (MAT) algorithm^34^. MAT is an innovative Transformer-based model designed for image inpainting, which combines the strengths of Transformers and convolutions to efficiently process high-resolution images. The algorithm excels in handling missing regions, analogous to the regions covered by the measurement markers in our ultrasound images. Specifically, in the first stage, the measurement markers are identified and segmented, effectively creating missing regions in the images where the markers were located. These missing regions are then treated as holes that need to be filled in by the MAT algorithm. MAT’s ability to handle missing regions makes it well-suited for this task, as it can generate plausible content for the areas where the measurement markers were removed, thereby restoring the integrity of the ultrasound images. This approach ensures that the regions of interest, such as tumors, are not affected by the presence of the measurement markers, leading to more accurate and reliable image analysis.

During the inference phase, the ultrasound images containing measurement markers are first processed by the segmentation model trained in the first stage to generate a segmentation mask. This segmentation mask is then used as input to the MAT model, which reconstructs the image by filling in the regions where the markers were located. This approach is advantageous over other methods, such as Generative Adversarial Networks, as it specifically targets the marker regions for generation, thereby preserving the integrity of the rest of the image.

By employing this two-stage approach, we are able to effectively remove measurement markers from ultrasound images, enhancing the quality of the images and reducing potential biases in subsequent analyses. Examples of the performance of de-marker model are presented in Supplementary Fig. 3. Following this process, a dedicated radiologist reviewed images in both the training and test sets to ensure removal of measurement markers, further reducing experimental bias. The comparative performance of UniMatch and LivNet on test sets with and without markers is detailed in Supplementary Table No. 2 and Supplementary Data 1.

### Development of the AI system

In this study, we systematically collected a robust training dataset, comprising a total of 17,913 ultrasound images, to quantitatively evaluate the performance of AI algorithms in ultrasound screening. This dataset served as the foundation for the development of two specialized AI models: UniMatch^35^, designed for the precise delineation of nodules within the images; and LivNet, tailored for the accurate classification of these nodules.

### Development of UniMatch

**Fig. 6:**
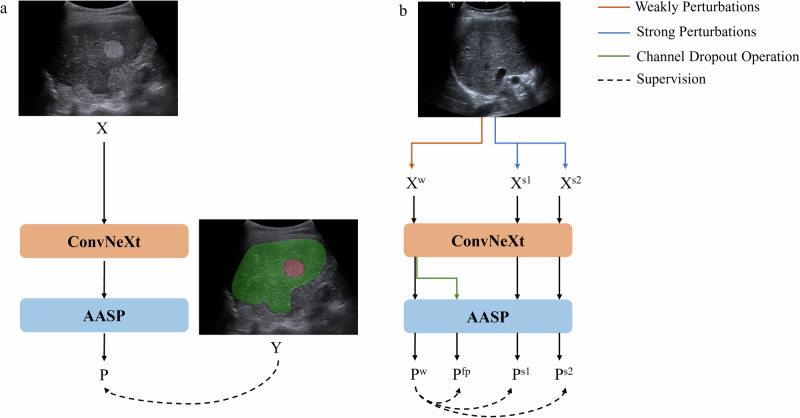
Network architecture of UniMatch. **a** The network architecture of labeled images. **b** The network architecture of unlabeled images. X = original images, Y = ground-truth mask of X, P = prediction of X, X^w^ = original image with weak perturbation, X^s1^ and X^s2^ = original images with strong perturbation, P^w^ = prediction of X^w^, P^fp^ = prediction of X^w^ with channel-wise dropout operation, P^s1^ and P^s2^= predictions of X^s1^ and X^s2^. AASP atrous spatial pyramid pooling.

For lesion detection, within training dataset, 1314 images were meticulously annotated for lesion segmentation using the *LabelMe* software (an open-source image annotation software). A semi-supervised semantic segmentation model, UniMatch (Fig. 6), was implemented to utilize both labeled and unlabeled data.

The UniMatch employs an encoder-decoder architecture, utilizing ConvNeXt as the encoder to effectively extract features from images, while employing Atrous Spatial Pyramid Pooling (ASPP) as the decoder to generate precise segmentation predictions.

For the labeled data, as demonstrated in the Fig. 6a, UniMatch adheres to a standard supervised learning approach. When provided with a labeled image-mask pair (X, Y), the framework optimizes its model by calculating the discrepancy between the predicted segmentation, P, and the ground-truth mask, Y. The discrepancy is quantified using the Cross-Entropy loss and the Intersection over Union (IoU) loss functions, as Eq. (1):

1 $${L}_{l}=-\mathop{\sum }\limits_{i=1}^{C}{Y}_{i}\,\log ({P}_{i})+\left(1-\frac{1}{C}\mathop{\sum }\limits_{i=1}^{C}\frac{{Y}_{i}{P}_{i}}{{Y}_{i}+{P}_{i}-{Y}_{i}{P}_{i}}\right)$$

where *C* is the total number of classes, *Y*_*i*_ and *P*_*i*_ represent the ground-truth label and predicted probability for the *i*-th class, respectively.

For each unlabeled image, UniMatch not only introduces conventional image-level perturbations to the input images but also extends these perturbations to the feature-level. As illustrated in the Fig. 6b, the encoder ConvNeXt first extracts the feature representation from the weakly augmented version X^w^ (e.g., resize, crop, flip), resulting in the prediction P^w^. Subsequently, a simple channel dropout operation (with a probability of 0.5) is applied to the latent feature space, leading to a perturbed feature representation. This perturbed feature is then processed by the decoder, ASPP, to yield the prediction P^fp^. Additionally, UniMatch creates two independent strong views (X^s1^ and X^s2^, X^s1^ ≠ X^s2^) by applying strong perturbations (e.g., color jitter, blur, grayscale, cutmix) to the weakly augmented image X^w^. These two strong views are processed in parallel to obtain their predictions (P^s1^ and P^s2^). The UniMatch component enhances the model’s capability to learn robust representations by enforcing consistency among predictions from weakly augmented images (P^w^), perturbed features (P^fp^), and strongly augmented images (P^s1^ and P^s2^). Specifically, P^w^ serves as a supervisory signal for P^fp^, P^s1^, and P^s2^. By minimizing the discrepancies between the predictions of P^w^, P^fp^, P^s1^, and P^s2^, the model is guided to learn representations that are resilient to image-level and feature-level perturbations, as Eq. (2):

2 $${L}_{u}=\mathop{\sum }\limits_{i=1}^{C}f(\max ({P}_{i}^{w})\ge \tau )\cdot \left\{CE({P}_{i}^{\,fp},{P}_{i}^{w})+CE({P}_{i}^{s1},{P}_{i}^{w})+CE({P}_{i}^{s2},{P}_{i}^{w})\right\}$$

In this context, *C* represents the total number of classes, $f(\max ({P}_{i}^{w})\ge \tau )$ represents the set of predictions in ${P}_{i}^{w}$ where the confidence exceeds the threshold *τ*(0.95), and *CE* denotes the Cross-Entropy Loss.

### Development of LivNet

**Fig. 7:**
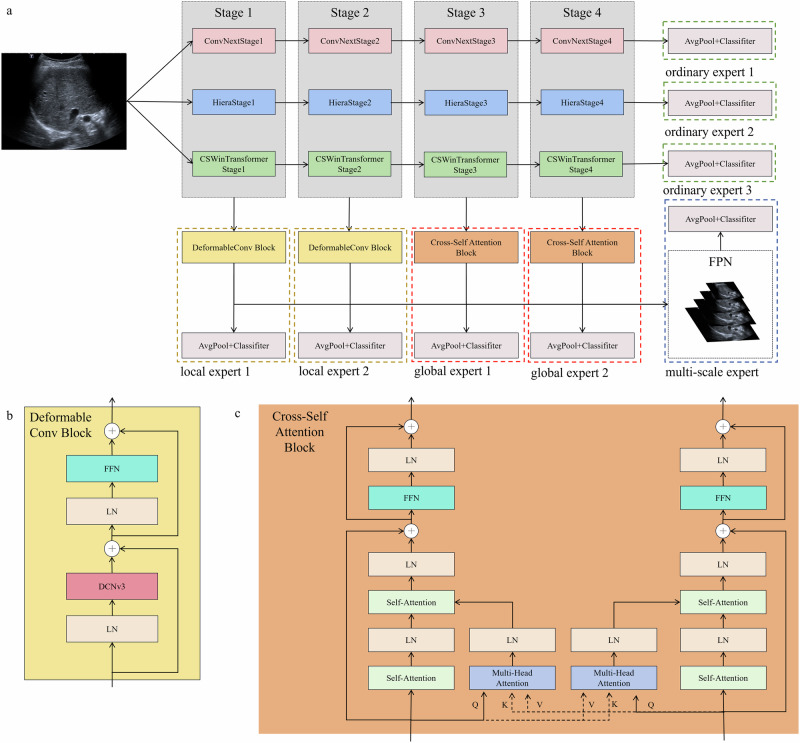
Network architecture of LivNet. **a** The blue dashed box represents the multi-scale expert module, the yellow dashed box represents the local expert module, the red dashed box represents the global expert module, and the green dashed box represents the ordinary expert module. The text below each dashed box indicates which expert it represents. **b** The network architecture of deformable convolution block. **c** The network architecture of cross-self attention block. FPN feature pyramid network. FFN feed-forward network. LN layer normalization.

To pursue accurate liver lesion classification, we have developed a robust hybrid model, LivNet (Fig. 7), which utilizes the training set comprised of 11,244 annotated images depicting benign and malignant liver lesions. This model innovatively combines the strengths of three state-of-the-art neural network architectures: ConvNeXt, CSWinTransformer and Hiera^36–38^. ConvNeXt excels at extracting intricate texture features, which are crucial for differentiating between various types of lesions. CSWinTransformer utilizes a cross-shaped window self-attention mechanism to effectively capture long-range dependencies and spatial configurations, thereby enhancing the model’s capacity to recognize complex patterns and relationships across the entire image. Meanwhile, Hiera’s hierarchical architecture captures input data across multiple semantic scales, enhancing the model’s ability to understand complex structures.

The three backbones of LivNet were initially trained separately on the ImageNet dataset, and were subsequently integrated using an innovative multi-expert hybrid fusion approach. This method encompasses four types of expert networks that are meticulously designed: the ordinary expert, the local expert, the global expert, and the multi-scale expert. These expert networks are designed to concentrate on feature-level interdependencies. The final prediction is generated through a dynamic, adaptive weighting mechanism that emphasizes decision-level correlations, which seamlessly integrates the outputs of expert networks to leverage their collective advantages to improve classification accuracy.

As illustrated in Fig. 7a, the design of the ordinary expert is straightforward, consisting of a Global Average Pooling (GAP) layer followed by a Fully Connected (FC) layer. This architecture aligns with standard backbone networks, utilizing the FC layer to directly classify high-level features extracted from deeper layers.

Local expert is designed for fine-grained recognition tasks. It consists of a deformable convolution module for adaptive feature extraction, coupled with a classification head for diagnostic prediction. The deformable convolution module comprises a 3 × 3 Deformable Convolution (DC) layer, two Layer Normalization (LN) layers and a Feed-Forward Network (FFN). The classification head uses GAP to aggregate features into a vector, which is classified by a FC layer for diagnosing fine-grained attributes. In this expert, features from the corresponding stages of three backbone networks are concatenated and then subjected to dimensionality reduction via a 1 × 1 convolution. Subsequently, an adaptive deformable convolution module is utilized to capture detailed texture information for the final classification diagnosis. This process can be summarized as Eqs. (3)–(5):

3 $${X}^{{\prime} }=DC(LN(X))+X$$

4 $$\tilde{X}=FFN(LN({X}^{{\rm{{\prime} }}}))+{X}^{{\rm{{\prime} }}}$$

5 $$Y=FC(GAP(\tilde{X}))$$

Global module comprises of a Cross-Self Attention (CSA) block and a classification head, with the CSA representing a novel attention mechanism developed in this work. The Self-Attention (SA) component is designed to capture dependency relationships and feature importance within individual backbone features, while the Cross-Attention (CA) component identifies correlations and dependencies among different backbone features. By integrating these two attention mechanisms in parallel, we can optimize their synergistic potential and amplify the individual strengths of SA and CA. Moreover, the repeated application of the SA can further enhance the model’s representational capacity and improve overall performance. Assuming that *x*_*scr*_ and *x*_*tgt*_ are the feature representations of the source sequence and the target sequence, respectively. Note that the global expert module is a symmetric structure. For simplicity, only one side of the situation is discussed here. The basic principle can be roughly described as follows, as Eqs. (6–8):

6 $$CSA({x}_{scr},{x}_{tgt})=SA(SA({x}_{scr})+CA({x}_{scr},{x}_{tgt}))$$

7 $$SA({x}_{scr})={x}_{scr}{W}_{Q}{({x}_{scr}{W}_{K})}^{T}{x}_{scr}{W}_{V}$$

8 $$CA({x}_{scr},{x}_{tgt})={x}_{scr}{W}_{Q}{({x}_{tgt}{W}_{K})}^{T}{x}_{tgt}{W}_{V}$$

where, *W*_*Q*_, *W*_*K*_, and *W*_*V*_ represent the weight matrices for query, key, and value, respectively.

Inspired by extensive findings^39–41^, we have developed a simplified multi-scale expert that incorporates a Feature Pyramid Network (FPN) module and a classification head. The FPN module aggregates output features from all preceding layers into a feature pyramid. In this pyramid, each level captures semantic information at different scales, ranging from high-resolution shallow features to low-resolution deep features. The concatenated multi-scale representations are then projected to the classification head for prediction. This design aims to bridge semantic gaps between layers and enable the network to jointly leverage cues at different scales for increased representation power.

The four types of expert modules previously discussed are designed to focus on feature-level correlations. To enhance the overall system performance, it is essential to effectively integrate the outputs of these individual modules, emphasizing decision-level correlations. In this section, we adopt a simplest weighted fusion approach, which involves assigning different weights to the outputs of each module based on their reliability or performance. This method leverages the principle that modules with higher reliability or accuracy should have a more significant influence on the final fused result. LivNet comprises 8 classification experts (Fig. 7a). Each expert is assigned a learnable parameter, *γ*, which determines the weight proportion of the expert’s prediction result. Throughout the training process, these weight parameters can be learned automatically to adaptively balance the predictive contributions of different experts (Supplementary Table No. 3).

### Test for human-AI interaction strategies

For the test set, 4021 images were included. There was no overlap between images in the training and test set. To ensure a valid and unbiased evaluation, the data split was conducted at the patient level, such that all images from a given patient were assigned exclusively to either the training or the test set. According to the original ultrasound screening procedure, both lesion detection and subsequent classification were performed by radiologists. In this study, four human-AI interaction strategies were simulated as follows: (a) Strategy 1 (Stand-Alone AI): UniMatch was used in lesion detection and LivNet was used in lesion classification to determine whether to recall or not; (b) Strategy 2 (AI as a triage tool with radiologist read negative cases in lesion detection): UniMatch was employed to detect lesions. If no lesion was detected, the image would be further evaluated by radiologists to ensure a high sensitivity of detection. After that, images without a lesion did not require recall. Images with lesions detected either by radiologists or UniMatch were classified by LivNet, benign lesions did not require recall, whereas malignancy lesions did; (c) Strategy 3 (AI as a triage tool and radiologist’s aid): UniMatch was utilized to detect lesions. If no lesion was detected, the image would be further evaluated by radiologists. After that, images without a lesion require no-recall. Images with lesions after recall were classified by radiologists assisted with LivNet; (d) Strategy 4 (AI as a triage tool with radiologist read negative cases in lesion detection and classification): UniMatch was used to detect lesions. If no lesion was detected, the image would be further evaluated by radiologists. After that, images without a lesion require no-recall. Images with lesions detected by radiologists or UniMatch were classified by LivNet. Lesions assigned as malignancy required recall, while those assigned as benign were further evaluated by radiologists to determine the necessity of recall, aiming to ensure a high sensitivity of classification (Fig. 1).

### Entropy

We calculated the Shannon entropy to quantify the uncertainty of prediction results for the four strategies. The formula used is:

9 $$H(X)=-\mathop{\sum }\limits_{i=1}^{n}p\left({x}_{i}\right){\log }_{2}\left(p\left({x}_{i}\right)\right)$$

*H (X)* represents the entropy, *X* is a discrete random variable indicating recall or no recall, *i* is an index variable, ${p(x}_{i}$) represents the probability of event ${x}_{i}$ occurring, and ${\log }_{2}$ is the logarithm base 2. The unit of entropy is bit. A higher entropy value indicates a higher uncertainty in the prediction results.

### Statistical analysis

All statistical analyses were conducted using Python version 3.8 (Python Software Foundation) and SPSS 22.0. The performance of models for lesion detection was evaluated by sensitivity, specificity, accuracy, and the area under the receiver operating characteristic curve (AUC). The performance of models for classification was assessed using sensitivity, specificity, accuracy and AUC. Screening workload was calculated as the number of images that radiologists need to read under different strategies. Recall rate was calculated as the percentage of total images necessitating a recall. False positive rate was calculated as the ratio between the number of negative images (no recall) wrongly categorized as positive (recall) and the total number of actual negative images. The screening performance of the original algorithm and the four strategies were assessed included sensitivity, specificity, accuracy, AUC, screening workload, recall rate and false positive rate. 95% confidence interval (CI) was calculated using the Clopper-Pearson method. Noninferiority in sensitivity was evaluated with a relative margin of 5%, and the lower limit of the 95% CI of the difference was greater than the negative value of a prespecified noninferiority margin was considered noninferior. Superiority in specificity was evaluated with an absolute margin of 1%, and the lower limit of the 95% CI of the difference was greater than the superiority margin was considered superiority. The chi-square test and z-test were used to assess differences in accuracy and screening workload between different strategy and original algorithm, respectively. The DeLong method was employed to determine significant differences between AUCs. Mann-Whitney U test was used for comparisons between different strategies. A significance level of α = 0.05 was applied to all comparisons. To account for multiple hypothesis testing, a correction with an adjusted α value of 0.05/n was applied, where n is the number of comparisons.

## Supplementary information

Supplementary Information

## Data Availability

The de-identified patient data may be made available upon reasonable request to the corresponding author, subject to approval from the Institutional Review Board. The code is available upon reasonable request to the corresponding author.
