# Supplementary material for: AI enhanced diagnostic accuracy and workload reduction in hepatocellular carcinoma screening
Source: NPJ Digit Med. 2025 Aug 2;8:500. doi: 10.1038/s41746-025-01892-9 (PMC12318110; doi:10.1038/s41746-025-01892-9)
Supplement: Supplementary file 1 — Supplementary Information [file 41746_2025_1892_MOESM1_ESM.pdf]

Supplementary Figure 1: Examples of the performance of UniMatch on the test set.

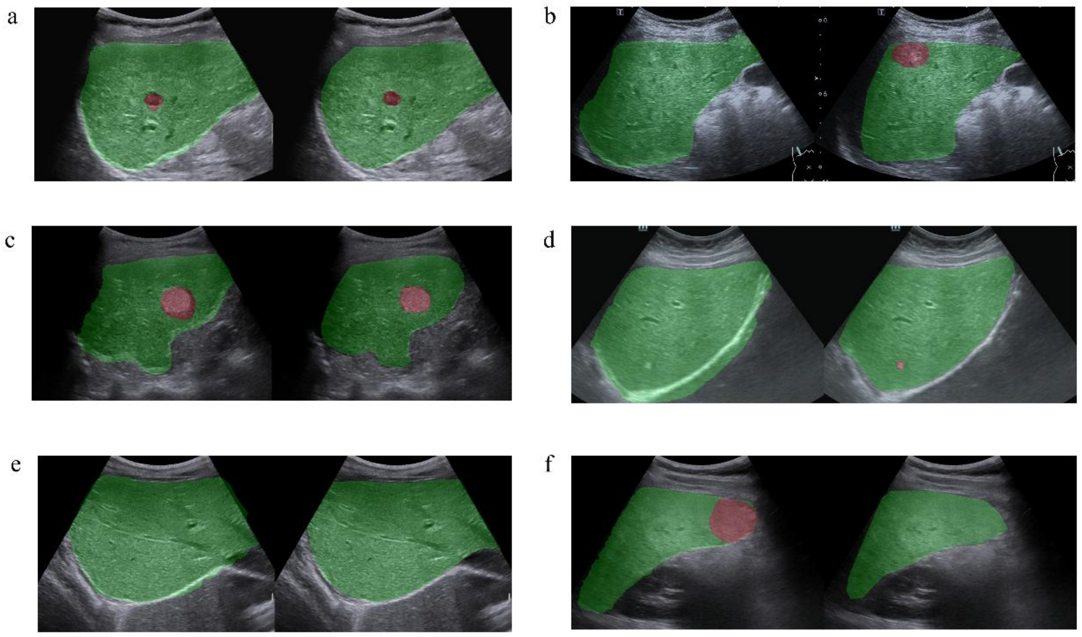

(a) Example of correctly predicted malignant lesion. (b) Example of incorrectly predicted malignant lesion. (c) Example of correctly predicted benign lesion. (d) Example of incorrectly predicted benign lesion. (e) Example of correctly predicted liver background. (f) Example of incorrectly predicted liver background.

Supplementary Figure 2: LivNet's visualization of heatmaps.

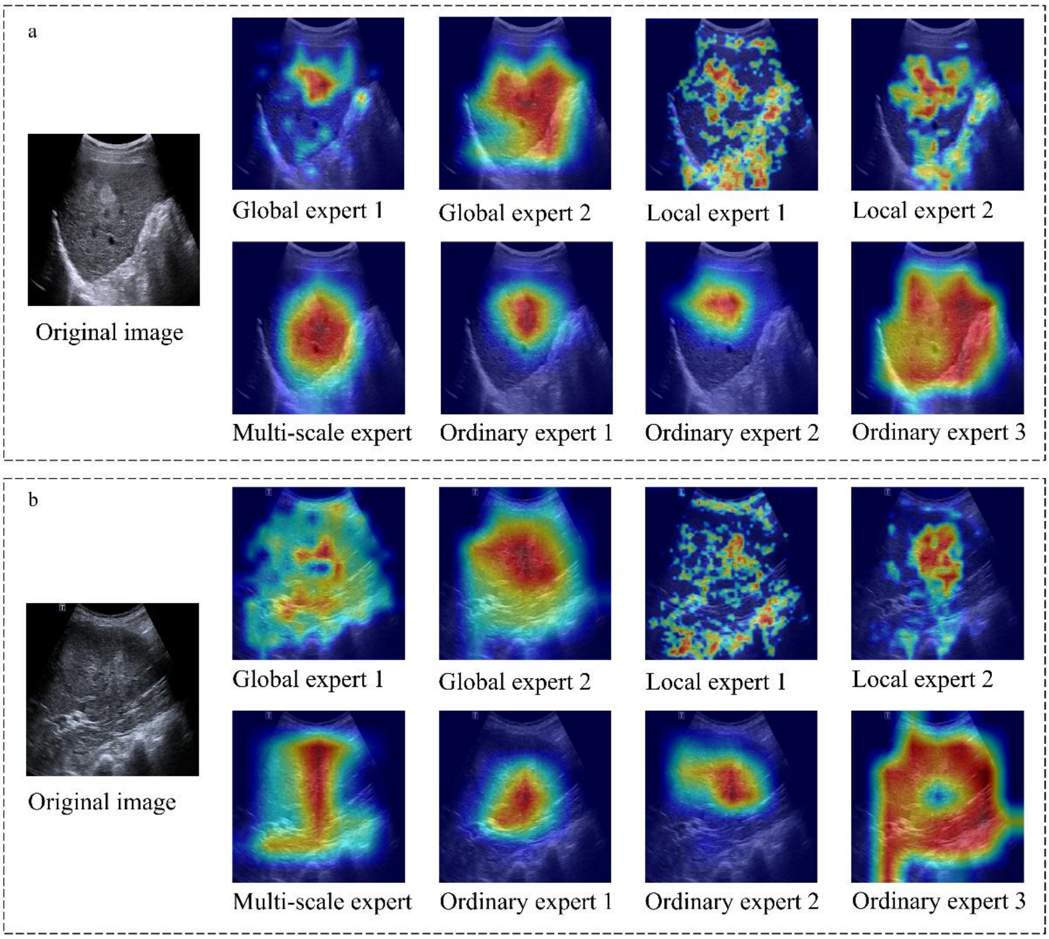

(a) The heatmaps visualization of the outputs from the 8 expert modules in the LivNet for a benign lesion. (b) The heatmaps visualization of the outputs from the 8 expert modules in the LivNet for a malignant lesion.

Supplementary Figure 3: Examples of the performance of de-marker model.

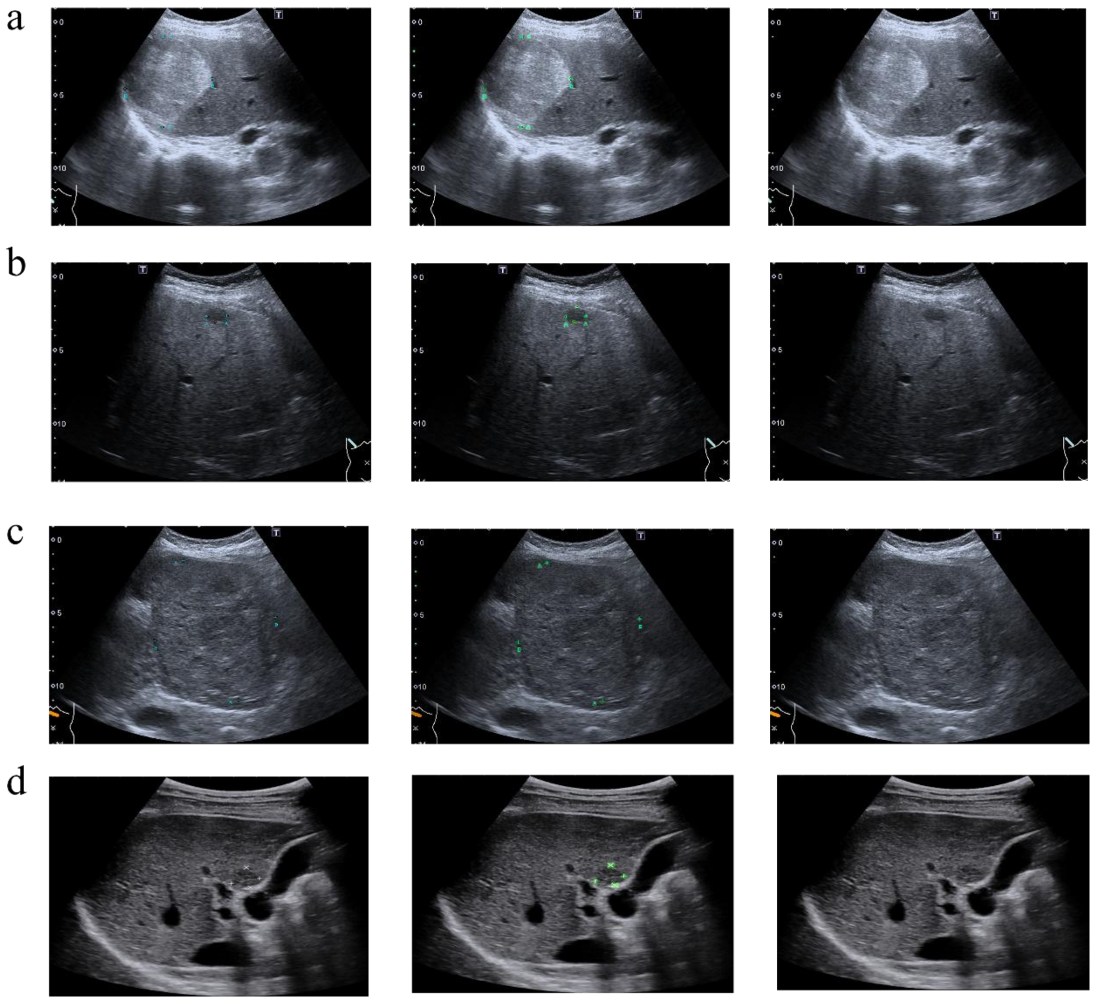

The leftmost column shows the original images with markers, the middle column displays the images with the markers identified and marked, and the rightmost column presents the images after the markers have been removed. (a) and (b) are examples of benign lesions. (c) and (d) are examples of malignant lesions.

Supplementary Table No. 1: Statistical results of lesion sizes for images with lesions that were incorrectly classified as lesion-free.

Parameter	Value
Mean ± SD	1.9 ± 1.4
Median	1.3
Max	8.9
Min	0.5

SD standard deviations.

Supplementary Table No. 2: Comparative performance of UniMatch and LivNet on marker-free and with-marker test sets.

Metric	UniMatch (marker-free)	UniMatch (with-markers)	P value	LivNet (marker-free)	LivNet (with-markers)	P value
Sensitivity	0.941 (0.932–0.949)	0.985 (0.980–0.989)	< 0.001	0.891 (0.877–0.903)	0.913 (0.901–0.925)	< 0.001
Specificity	0.833 (0.808–0.855)	0.817 (0.792–0.8841)	< 0.001	0.783 (0.762–0.802)	0.704 (0.681–0.725)	< 0.001
Accuracy	0.914 (0.905–0.923)	0.944 (0.936–0.950)	< 0.001	0.844 (0.832–0.855)	0.823 (0.811–0.835)	< 0.001
AUC	0.887 (0.876–0.896)	0.901 (0.892–0.910)	< 0.001	0.837 (0.825–0.848)	0.808 (0.796–0.821)	< 0.001

AUC area under the receiver operating characteristic curve.

Supplementary Table No. 3: Weight distribution of eight classification experts in LivNet.

	Multi-scale expert	Local expert 1	Local expert 2	Global expert 1	Global expert 2	Ordinary expert 1	Ordinary expert 2	Ordinary expert 3
Weight	0.078	0.272	0.205	0.157	0.183	0.086	0.016	0.003

Supplementary Data 1: Analysis of model performance on rest sets with and without markers.

We conducted a stratified analysis to evaluate model performance separately on the test set originally containing measurement markers and on the test set without markers. The complete results are shown in Supplementary Table 2.

For the test set with markers, UniMatch achieved a sensitivity of 0.985 (95% CI: 0.980–0.989), specificity of 0.817 (0.792–0.8841), accuracy of 0.944 (0.936–0.950), and an AUC of 0.901 (0.892–0.910). In this set, 183 images without lesions were incorrectly classified as containing lesions, while 44 images with lesions were missed. On the test set without markers, UniMatch reached a sensitivity of 0.941 (95% CI: 0.932–0.949), specificity of 0.833 (0.808–0.855), accuracy of 0.914 (0.905–0.923), and an AUC of 0.887 (0.876–0.896). 180 lesion-free images were falsely identified as having lesions, and 165 images with lesions were misclassified as lesion-free. McNemar's tests for all metrics showed statistically significant differences ($p < 0.001$).

For LivNet, on the test set with markers, sensitivity, specificity, accuracy, and AUC were 0.913 (95% CI: 0.901–0.925), 0.704 (0.681–0.725), 0.823 (0.811–0.835), and 0.808 (0.796–0.821), respectively. On the test set without markers, these metrics were

0.891 (95% CI: 0.877–0.903), 0.783 (0.762–0.802), 0.844 (0.832–0.855), and 0.837 (0.825–0.848), with all differences statistically significant ($p < 0.001$).

Compared to the marker-free test set, the test set originally containing markers resulted in significantly higher sensitivity, accuracy, and AUC for UniMatch, accompanied by a slight decrease in specificity ($p < 0.001$). This is likely because the presence of markers provided guidance that facilitated target localization. In contrast, LivNet exhibited increased sensitivity but decreased specificity, accuracy, and AUC ($p < 0.001$), possibly due to being trained exclusively on marker-free images and the presence of markers during testing partially obscured the image, disrupting the overall image distribution and impairing classification performance
